# Influence of Oxygen-Release Material Doping on the Optical Properties of La_1–x_Sr_x_TiO_3+δ_

**DOI:** 10.3390/ma18112553

**Published:** 2025-05-29

**Authors:** Wenzhi Li, Yichao Zhu, Zhiping He

**Affiliations:** China Helicopter Research and Development Institute, Jingdezhen 333001, Chinahezp@avic.com (Z.H.)

**Keywords:** optical property, oxygen vacancy suppression, La_1–x_Sr_x_TiO_3+δ_, oxygen-release doping, valence state

## Abstract

This study focuses on addressing the reflectivity reduction issue in La_1–x_Sr_x_TiO_3+δ_ during high-temperature preparation, which is caused by oxygen vacancy generation. Bulk samples of CeO_2_-doped La_1–x_Sr_x_TiO_3+δ_ with varying doping contents as a second phase and sintering temperatures were prepared. The phase composition, reflectivity, and valence states were thoroughly investigated. Introducing 10 wt.%CeO_2_ significantly suppressed the formation of oxygen vacancies. Thus, the occurrence of impurity levels caused by oxygen vacancies was reduced. This can further mitigate the reflection decrease caused by impurity levels as photon absorption traps. Additionally, the reduced pore structure achieved at 1450 °C contributed to improved reflectivity compared to pure La_1–x_Sr_x_TiO_3+δ_. The findings suggest that this approach has great potential for reducing oxygen vacancies sensitivity in high-reflection ceramics under high-temperature conditions and preserving their optical properties.

## 1. Introduction

La_1–x_Sr_x_TiO_3+δ_ (LST) is a perovskite-type oxidation that has garnered significant attention due to its promising applications in various fields, particularly in optics and electronics. This material is particularly noted for its unique electrical and optical properties, which make it suitable for a range of advanced technological applications [1,2,3,4]. The material’s ability to maintain good optical transparency over a wide range of wavelengths is a desirable feature for such applications [2]. In addition, the reflectivity of LST is also of interest. The research of Zhu indicates that LST exhibits high reflectivity. Given its high thermal stability, LST can be potentially applied as a high-reflection material [3]. However, some studies have reported that oxygen vacancies are easily formed and result in an LST material system when the LST material is prepared at a low oxygen partial pressure or in a high-temperature environment [4,5,6]. Meanwhile, the generation of oxygen vacancy leads to a reflectivity reduction in LST. This phenomenon limits the further application of LST in some particular conditions, such as optical material [7,8].

The performance of ceramic materials is often significantly influenced by doping, which serves as a critical approach to enhancing their thermal, optical, mechanical and other properties [9,10,11,12]. Doping involves the intentional introduction of foreign ions into the crystal lattice of a base material, leading to modifications in its electronic structure, defect chemistry, and surface characteristics. These changes can result in improved stability under extreme conditions, enhanced catalytic activity, or tailored optical responses, depending on the specific application requirements.

Among various dopants, CeO_2_ stands out as a particularly promising candidate due to its unique properties. CeO_2_ is a well-known oxygen storage material with a cubic fluorite structure that allows for a high concentration of surface oxygen vacancies [13,14]. These oxygen vacancies are not only capable of storing and releasing oxygen but also provide active sites for catalytic reactions [15,16,17]. The ability of CeO_2_ to reversibly store and release oxygen makes it an attractive candidate for applications in solid oxide fuel cells, catalysis, and gas sensing [18,19]. Given the aforementioned characteristics of CeO_2_, doping LST with CeO_2_ presents an innovative solution. Specifically, the addition of CeO_2_ could enhance the oxygen storage capacity of LST, thereby compensating for its inherent oxygen deficiency. Additionally, the presence of CeO_2_ may influence the optical properties of LST.

The optimization of doping levels and conditions is crucial to achieving the desired enhancements in performance. For example, excessive doping could lead to the formation of secondary phases or disrupt the lattice structure of LST, thereby compromising its mechanical integrity. On the other hand, insufficient doping may fail to deliver the expected improvements in oxygen storage capacity or optical properties. Therefore, a systematic investigation is required to determine the optimal concentration and distribution of CeO_2_ within the LST matrix.

In summary, the strategic doping of LST with CeO_2_ as a second phase offers a promising avenue for overcoming its inherent limitations. In this study, in order to suppress oxygen vacancy generation and maintain the high reflection of LST, the phase, reflectivity, and valence states of CeO_2_-doped samples with different content and different sintering temperatures were investigated in depth.

## 2. Materials and Methods

### 2.1. Sample Preparation

A high-temperature solid-state reaction method was used to prepare the samples with high-purity raw materials: La_2_O_3_ (99.99%, Forsman Scientific Co., Ltd., Beijing, China), SrCO_3_ (AR, Forsman Scientific Co., Ltd., Beijing, China), TiO_2_ (AR, Forsman Scientific Co., Ltd., Beijing, China), and CeO_2_ (AR, Forsman Scientific Co., Ltd., Beijing, China). These raw materials of the LST matrix were weighed stoichiometrically according to the previous work [1]. The proportions of LST and CeO_2_ were weighed and sintered at the corresponding temperature according to the formulation listed in Table 1.

### 2.2. Characterization

X-ray diffraction (PANalytical Inc., X’Pert PRO MPD, Almelo, The Netherlands) was used to characterize the phase transition of samples with different proportions and the results were analyzed by JADE (version 5.0, Materials Data Inc., Livermore, CA, USA). The surface microstructure of sintered samples was observed by a scanning electron microscope (HITACHI S4800, HITACHI, Tokyo, Japan). The reflectivity of every sample was measured by an ultraviolet–visible–near-infrared (UV–VIS–NIR) spectrophotometer (Cray5000, Palo Alto, CA, USA). Meanwhile, the valence states of Ti and Ce were tested by X-ray photoelectron spectroscopy (PHI 5300, PHI, Lafeyette, LA, USA) in order to explore the optic property changes.

## 3. Results and Discussion

### 3.1. Surface Morphology and Optical Analysis of Different CeO_2_ Content-Doped LST-CO

**Figure 1:**
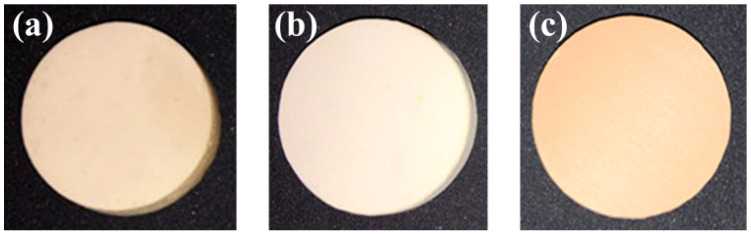
Surface macro-morphologies of (**a**) LC0, (**b**) LC1, and (**c**) LC2 samples.

The surface macro-morphologies of different CeO_2_-doped LST bulks (LC0, LC1, and LC2) are shown in Figure 1. A visible color difference can be observed with different CeO_2_-doped LST bulks sintered at 1450 °C. With the 20 wt.% CeO_2_ doping, the sintered bulk exhibits a significant color change. According to the experience obtained in a previous study, light-colored samples may exhibit a higher reflectivity [3].

**Figure 2:**
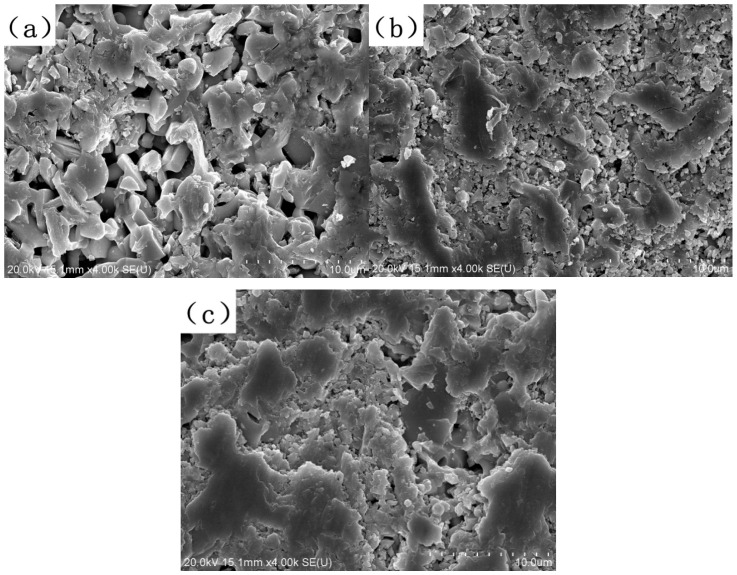
Surface microstructure of (**a**) LC0, (**b**) LC1, and (**c**) LC2 samples.

Additionally, when observing the surface microstructures of LC0, LC1, and LC2 bulks shown in Figure 2, LC1 and LC2 exhibit less visible pores compared to LC0, which means less absorption will be induced by the porous structure.

From the energy band theory, the optical properties of materials are influenced by the transition and return of electrons between the conduction band and valence band. The appearance of oxygen vacancies leads to the generation of impurity levels below the conduction band. The distance of the generated new impurity levels is less than the bandgap of crystal without oxygen vacancies. Therefore, the subbands behave as trap centers inside the bandgap of the ideal case [20,21]. Some incident photons are absorbed rather than reflected. This is the mechanism of the influence of oxygen vacancy generation on reflectivity.

**Figure 3:**
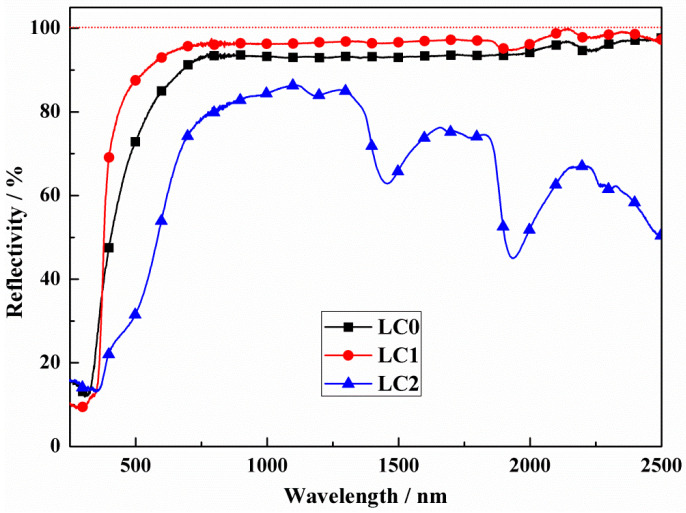
Reflectivity of LC0, LC1, and LC2 samples.

On the basis of the reflectivity spectra shown in Figure 3, the above conjecture that LC1 bulks have the highest reflectivity has been confirmed. THe LC0 and LC1 bulks show the same variation trend with an obvious reflection from 800 nm to 2500 nm, and the maximum values can reach 92% and 96% at 1064 nm, respectively. The doping of CeO_2_ does play a positive role in enhancing the reflectivity of LST-CO. The appropriate CeO_2_ doping should have reduced the generation of oxygen vacancies in LST. This phenomenon also reduced the generation of impurity levels and increased the reflectivity of the sample. However, the highest reflectivity of LC2 is just 80% and a dramatic decrease appears around 2000 nm. This phenomenon may be because at a 20 wt.% doping ratio, the released oxygen of CeO_2_ escaped as O_2_ rather than being captured by the LST oxygen vacancies. These impurity levels of oxygen vacancies play a role in absorbing incident photons and preventing reflection [7].

### 3.2. Surface Morphology and Optical Property Analysis of Different-Temperature-Sintered LST-CO

**Figure 4:**
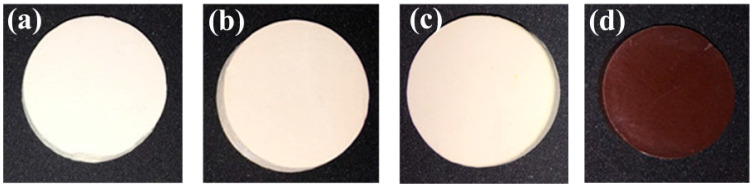
Surface macro-morphologies of (**a**) LC5, (**b**) LC4, (**c**) LC1, and (**d**) LC3 samples.

**Figure 5:**
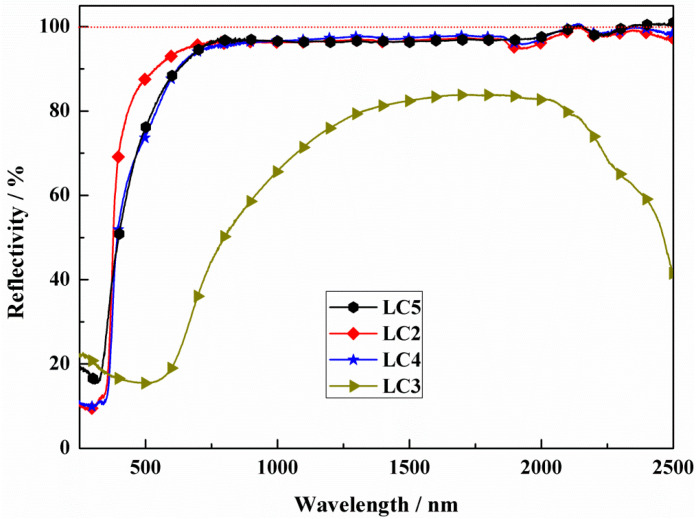
Reflectivity of LC2, LC3, LC4, and LC5 samples.

Furthermore, a series LST-CO sample with 10 wt.% CeO_2_ was prepared at different temperatures to further investigate the inhibitory effect of temperature on the generation of oxygen vacancy in LST. Their surface macro-morphologies are listed in Figure 4. With the increase in sintering temperature, the color of the sample surface gradually darkens. Combined with the reflectance spectra shown in Figure 5, it can be seen that there is no obvious difference in reflectivity at 1064 nm of the LC5, LC4, and LC1 samples when the processing temperature is below 1450 °C. Nevertheless, at an ultra-high sintering temperature (1550 °C), a significant decrease in reflectivity appears in the LC3 sample. The appearance of this phenomenon is because at ultra-high temperatures, a large amount of oxygen vacancies are generated and the induced impurity levels dominate the return of photons to the bandgap.

**Figure 6:**
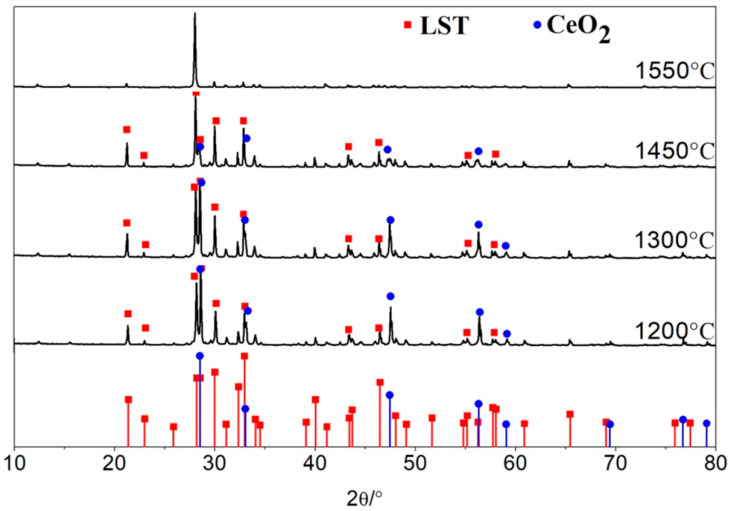
XRD patterns of LC2, LC3, LC4, and LC5 samples.

According to the XRD pattern shown in Figure 6, the phase structures of LST and CeO_2_ can be easily detected below 1450 °C, indicating that the LST and CeO_2_ have good thermal stability and no other new phase transform. However, some diffraction peaks at 1550 °C become weaker than those at any other low temperature, which can be explained by two aspects. On one hand, at that high temperature, a solid solution reaction of CeO_2_ and LST occurs, meaning that Ce^4+^ could replace the Ti^3+^ in LST. On the other hand, a long high-temperature sintering time causes grain growth and grain boundary reduction [22].

**Figure 7:**
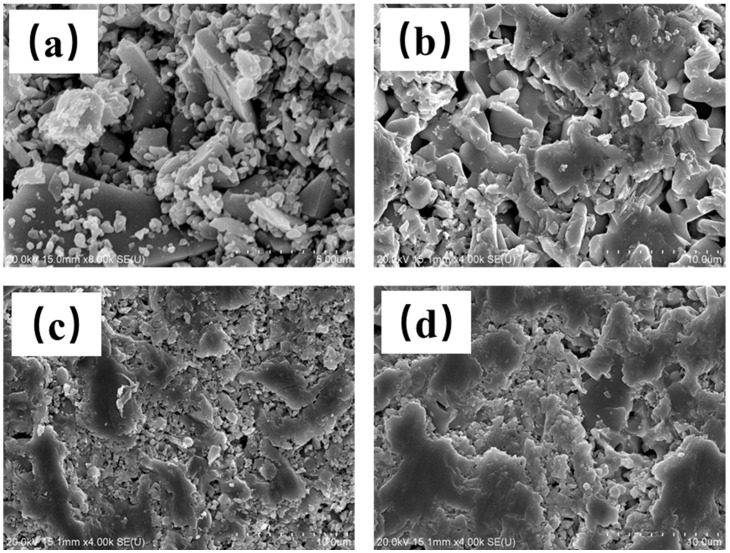
Surface microstructure of (**a**) LC5, (**b**) LC4, (**c**) LC1, and (**d**) LC3 samples.

In addition, from the microstructure of different-temperature-sintered LST-CO samples shown in Figure 7, the density of the sample shows an obvious enhancement with the increase in temperature. Considering that the mechanical properties of ceramics are positively correlated with their density [23], the material ratio and preparation parameters of the LC2 sample have good potential to improve the application of LST in some special fields with high reflection.

### 3.3. Phase and XPS Analysis of Different-Temperature-Sintered LST-CO

**Figure 8:**
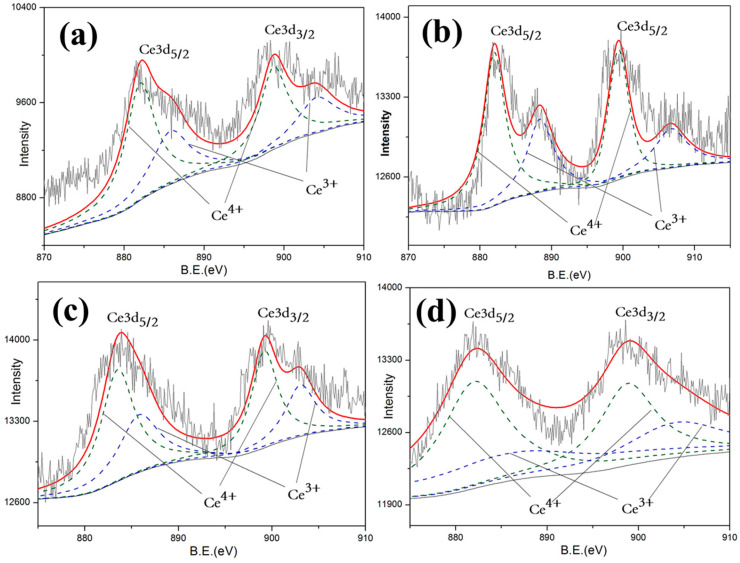
Total Ce3d spectroscopy of LST-CO samples. (**a**) LC5, (**b**) LC4, (**c**) LC1, and (**d**) LC3.

**Figure 9:**
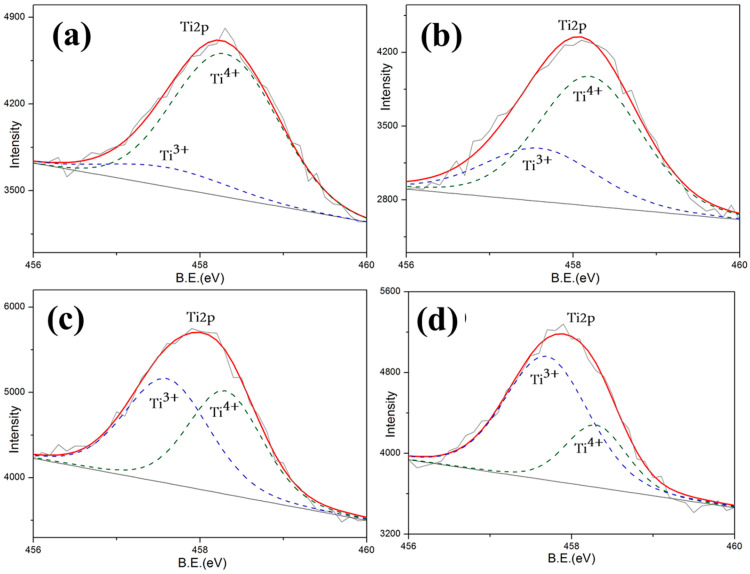
Total Ti2p spectroscopy of LST-CO samples. (**a**) LC5, (**b**) LC4, (**c**) LC1, and (**d**) LC3.

In order to further investigate the mechanism of the optical properties exhibited above, in-depth research into chemical valence was preceded by X-ray photoelectron spectroscopy (XPS) of 10 wt.% CeO_2_-doped LST. By analyzing the Ti2p and Ce3d energy bands of samples shown in Figure 8 and Figure 9, the valence states of Ti and Ce can be detected. Since the oxygen vacancy drives the Ti valence state from Ti^4+^ to Ti^3+^ and also drives the Ce valence state from Ce^4+^ to Ce^3+^ by giving additional electrons, the variation in oxygen vacancy can be calculated by the content of Ti^4+^ and Ce^4+^.

The valence state statistics of 10%wt. CeO_2_-doped LST sintered under different temperatures are shown in Table 1. The valence state variation in Ti and Ce form 4+ to −3+, shown in Reactions (1) and (2), which provides indirect evidence of the formation of oxygen vacancies shown in Reaction (3). According to the law of conservation, the more that reaction (2) occurs, the less reaction (1) occurs. Since the △H of reaction (2) is much lower than that of reaction (1), reaction (2) is more likely to happen at high temperatures. Thus, the number of oxygen vacancies generated in LST is reduced. LST can also obtain the oxygen from CeO_2_ to slow down the generation of its own oxygen vacancies.

Ti^4+^ + e → Ti^3+^ ∆H = −3.86kJ/mol (1)

Ce^4+^ + e → Ce^3+^ ∆H = −155.4kJ/mol (2)

O^2−^→0.5O_2_ + 2e + Vo (oxygen vacancy) (3)

More Ti^4+^ and Ce^4+^ maintaining shown in Table 2 indicates that fewer oxygen vacancies are generated. Oxygen vacancies generated at high temperatures act as absorption centers during the propagation of the light within the crystal, and light phonon scattering easily occurs in crystal structural defects [24,25,26,27]. This result proves that the reason for the dramatic decrease at 1550 °C is the generation of a large amount of oxygen vacancies. On the contrary, in addition to the contribution of the reduced pores of the LC1 structure, it shows a good reflection and high preparation temperature. This is consistent with the reflectivity test results shown in Figure 5.

According to the research results, it can be concluded that the improvement of the reflectivity of LST by CeO_2_ doping as a second phase depends on the doping content and preparation temperature. These two factors directly affect the style of oxygen released by CeO_2_, either as O_2_ or captured by LST (oxygen release and capture strategy). Based on all measurements, the sample with 10 wt.% CeO_2_ doping and sintering at 1450 °C achieved the best optical properties. The trend is shown in Table 3. Compared to other related work, although we did not find the same strategy, the work by Zhao et al. elaborated on another feasible approach to reducing the generation of oxygen vacancies by utilizing multi-element doping [28]. This method increases the generation energy of oxygen vacancies, which can mitigate the impact of oxygen vacancies on optical properties. This strategy enables the sustainment of material reflectance (92% at elevated sintering temperatures (1500 °C). These two methods have successfully suppressed the influence of oxygen vacancies on reflective performance. In the future, we will continue to investigate the feasibility of applying our “oxygen release and capture strategy” to high-temperature and high-reflection coatings/films. Meanwhile, we will also use DFT modeling and first-principles calculation methods to perform a deep investigation.

## 4. Conclusions

The bulk samples of CeO_2_-doped LST with different doping contents and sintering temperatures were prepared to investigate the effects of CeO_2_ doping as a second phase on the oxygen-deficient properties and reflection behavior of LST under high-temperature conditions.

Through systematic experiments, it was found that increasing the doping content of CeO_2_ led to a decrease in reflectivity due to the preferential release of oxygen as O2 rather than capturing by LST oxygen vacancies when the CeO_2_ doping content was over 10 wt.%. This indicates that the efficiency of oxygen release is higher than oxygen capture in this system. Under 10 wt.% CeO_2_ doping, the reflectivity exhibits a mountain peak-like trend with increasing preparation temperature. The sample at 1450 °C exhibits the best reflection property, up to 96%. At ultra-high temperatures (1550 °C), the generation of a large number of oxygen vacancies reduces reflection. However, small amounts of CeO_2_ doping can effectively improve oxygen deficiency and enhance reflection under high-temperature preparation conditions.

The findings demonstrate that CeO_2_ doping significantly influences the oxygen-related properties and optical behavior of LST at elevated temperatures. This provides valuable insights into designing advanced materials for applications requiring stable oxygen-deficient states and controlled reflectivity. The results suggest potential pathways for optimizing LST-based coatings or devices operating in high-temperature environments. This material can be applied in optical protective fields with high temperature requirements.

## Figures and Tables

**Table 1:** The formulation of LST-CeO_2_ (LST-CO) samples.

Sample	Temperature/°C	LST/wt.%	CeO2/wt.%
LC0	1450	100	0
LC1	1450	90	10
LC2	1450	80	20
LC3	1550	The appropriate proportion of LST and CeO_2_	
LC4	1300		
LC5	1200		

**Table 2:** Valence states of Ti and Ce at different sintering temperatures.

Sample (Sintering Temperature °C)	Ti^4+^/%	Ti^3+^/%	Ce^4+^/%	Ce^3+^/%	Total of Ti^4+^ and Ce^4+^
LC5 (1200)	86.6	13.4	63.8	36.2	150.4
LC4 (1300)	73.3	26.7	63.3	36.7	136.6
LC1 (1450)	68.8	31.2	61.6	38.4	130.4
LC3 (1550)	26.8	73.2	46.9	53.1	73.7

**Table 3:** Reflectivity of LST-CO samples under 1064 nm.

	Doping Content/wt.%	0	10	20
Temperature/°C				
1200		-	97	-
1300		-	96	-
1450		92	96	83
1550		-	66	-

## Data Availability

The original contributions presented in this study are included in the article. Further inquiries can be directed to the corresponding author.
